# The Physiological Functions and Structural Determinants of Catalytic Bias in the [FeFe]-Hydrogenases CpI and CpII of *Clostridium pasteurianum* Strain W5

**DOI:** 10.3389/fmicb.2017.01305

**Published:** 2017-07-12

**Authors:** Jesse B. Therien, Jacob H. Artz, Saroj Poudel, Trinity L. Hamilton, Zhenfeng Liu, Seth M. Noone, Michael W. W. Adams, Paul W. King, Donald A. Bryant, Eric S. Boyd, John W. Peters

**Affiliations:** ^1^Department of Chemistry and Biochemistry, Montana State University, Bozeman MT, United States; ^2^Department of Microbiology and Immunology, Montana State University, Bozeman MT, United States; ^3^Department of Biochemistry and Molecular Biology, The Pennsylvania State University, University Park PA, United States; ^4^National Renewable Energy Laboratory, Biosciences Center, Golden CO, United States; ^5^Department of Biochemistry & Molecular Biology, University of Georgia, Athens GA, United States

**Keywords:** hydrogenase, nitrogenase *Clostridium pasteurianum*, hydrogen metabolism, nitrogen metabolism, CpI, CpII

## Abstract

The first generation of biochemical studies of complex, iron-sulfur-cluster-containing [FeFe]-hydrogenases and Mo-nitrogenase were carried out on enzymes purified from *Clostridium pasteurianum* (strain W5). Previous studies suggested that two distinct [FeFe]-hydrogenases are expressed differentially under nitrogen-fixing and non-nitrogen-fixing conditions. As a result, the first characterized [FeFe]-hydrogenase (CpI) is presumed to have a primary role in central metabolism, recycling reduced electron carriers that accumulate during fermentation via proton reduction. A role for capturing reducing equivalents released as hydrogen during nitrogen fixation has been proposed for the second hydrogenase, CpII. Biochemical characterization of CpI and CpII indicated CpI has extremely high hydrogen production activity in comparison to CpII, while CpII has elevated hydrogen oxidation activity in comparison to CpI when assayed under the same conditions. This suggests that these enzymes have evolved a catalytic bias to support their respective physiological functions. Using the published genome of *C. pasteurianum* (strain W5) hydrogenase sequences were identified, including the already known [NiFe]-hydrogenase, CpI, and CpII sequences, and a third hydrogenase, CpIII was identified in the genome as well. Quantitative real-time PCR experiments were performed in order to analyze transcript abundance of the hydrogenases under diazotrophic and non-diazotrophic growth conditions. There is a markedly reduced level of CpI gene expression together with concomitant increases in CpII gene expression under nitrogen-fixing conditions. Structure-based analyses of the CpI and CpII sequences reveal variations in their catalytic sites that may contribute to their alternative physiological roles. This work demonstrates that the physiological roles of CpI and CpII are to evolve and to consume hydrogen, respectively, in concurrence with their catalytic activities *in vitro*, with CpII capturing excess reducing equivalents under nitrogen fixation conditions. Comparison of the primary sequences of CpI and CpII and their homologs provides an initial basis for identifying key structural determinants that modulate hydrogen production and hydrogen oxidation activities.

## Introduction

The genus *Clostridium* includes a diverse group of Gram-positive, spore-forming anaerobes (Patakova et al., 2013). In general, clostridial fermentative metabolism functions by the conversion of hexose sugars to butyrate, acetate, and CO_2_. During this process reduced electron carriers in the form of ferredoxin accumulate and must be recycled for sustained fermentative energy metabolism. *C. pasteurianum* recycles reduced ferredoxin by coupling electrons and protons to form hydrogen (H_2_) through the activity of a hydrogenase. *C. pasteurianum* may also fix nitrogen during fermentative growth, a process that requires high amounts of both ATP and reducing equivalents (Mortenson, 1964).

*Clostridium pasteurianum* strain W5 has been a model for studying the biochemistry of nitrogen fixation and H_2_ metabolism. The first preparations of a soluble hydrogenase (CpI) were obtained from this organism (Valentine et al., 1963), and subsequently, the presence of a second [FeFe]-hydrogenase (CpII) was revealed (Chen and Blanchard, 1978), and its physical and catalytic properties were studied along with those of CpI (Adams and Mortenson, 1984a).

[FeFe]-hydrogenase 1 from CpW5 was proposed to evolve H_2_ to recycle electron carriers during fermentative growth in the presence of fixed nitrogen (Adams and Mortenson, 1984a). CpII was proposed to function under nitrogen-fixing conditions to capture reducing equivalents in the form of H_2_ which is an obligate byproduct of nitrogenase-catalyzed reduction of nitrogen to ammonia. This is consistent with the observations that CpII accumulates at a higher cellular concentration during diazotrophic growth (Chen and Blanchard, 1978). Comparison of the rates of H_2_ evolution and oxidation revealed that, while these two enzymes are both reversible *in vitro*, CpI produces H_2_ 550-times faster than CpII (5,500 vs. 10 μmol of H_2_/min⋅mg, respectively) while it oxidizes H_2_ about 30% slower than CpII (24,000 vs. 34,000 μmol of H_2_/min⋅mg, respectively) (Adams, 1990). Typically, [FeFe]-hydrogenases have characteristically high catalytic rates for H_2_ production (Adams, 1990). Therefore, the two [FeFe]-hydrogenases exhibit a strong “catalytic bias,” which is manifested as the adaptation of CpII toward H_2_ oxidation.

Hydrogenases have been characterized as providing many functions *in vivo*, including disposal of excess reducing equivalents (Verhagen et al., 1999; Tamagnini et al., 2007; Schut and Adams, 2009), hydrogen sensing (Lenz et al., 2002; Brecht et al., 2003), ion translocation (Hedderich, 2004), and oxidizing H_2_ (Dementin et al., 2004; Kanai et al., 2011), a key method by which reducing equivalents are generated. This diversity of function allows hydrogenases to perform a critical role in a variety of metabolisms, including those of methanogens (Thauer et al., 2010), sulfate reducers (Baltazar et al., 2011), nitrogen fixers (Zhang et al., 2014), and photosynthesizers (Melis et al., 2004; Ghirardi et al., 2006; Tamagnini et al., 2007).

In this work we provide experimental evidence that under nitrogen replete conditions (in the absence of nitrogenase), CpI functions to reduce protons during the recycling of electron carriers during fermentation, while CpII functions in H_2_ oxidation under diazotrophic conditions. The genome of *C. pasteurianum* ATCC 6013 (strain W5) (Rotta et al., 2015) was subjected to homology searches using known hydrogenase sequences as queries to determine the complement of encoded hydrogenases, their sequences and their gene context. Using these data, we analyzed the transcript abundance of each hydrogenase under nitrogen-fixing and nitrogen-replete culture conditions to assign physiological roles for CpI and CpII. Furthermore, detailed primary amino acid structural-based comparison together with phylogenetic analysis provide insights into the determinants of the profound catalytic bias observed for these two related enzymes.

## Results and Discussion

### Genome

The sequencing of the *C. pasteurianum* strain W5 (CpW5) genome was carried out independently of the recently published complete genome (Rotta et al., 2015). Our analysis resulted in a draft genome consisting of 14 contigs and 4.2 Mbp that shares 99.97% average nucleotide identity with the published genome (Supplementary Figure 1). The published complete genome contains 4.3 Mbp, which indicates that our genome is nearly complete. In particular, the sequences of the genes encoding all four hydrogenases discussed in the present study are identical to those in the complete genome (Rotta et al., 2015). Like the genomes of other clostridial species (Sakaguchi et al., 2005; Yutin and Galperin, 2013; Sedlar et al., 2015), the GC content of CpW5 was low at 30.0%. *C. pasteurianum* NRRL B-598, which is an oxygen-tolerant species, is also related to CpW5 and has a genome size that is ∼50% larger, comprising 6.1 Mbp (Kolek et al., 2014). According to SEED Viewer (Overbeek et al., 2014), which does not include sequences from these *C. pasteurianum* genomes (i.e., ATCC 6013 DSM 525 and NRRL B-598), the closest neighbors with completed genomes are *C. acetobutylicum* (3.94 Mbp) (Nolling et al., 2001), *C. botulinum* (3.89 Mbp) (Sebaihia et al., 2007), *C. novyi* NT (2.55 Mbp) (Bettegowda et al., 2006), and *C. sporogenes* ATCC 15579 (4.09 Mbp) (Poehlein et al., 2015).

### Hydrogenases

The genome of CpW5 encodes the two characterized [FeFe]-hydrogenases, CpI and CpII, and an additional homolog designated CpIII, as well as one (previously annotated) [NiFe]-hydrogenase (Pyne et al., 2014), together with all of the necessary genes for hydrogenase maturation. These sequence data therefore allow us to carry out the first comparative analysis of the primary sequence of CpII since it was biochemically characterized more than two decades ago (Adams and Mortenson, 1984a).

**FIGURE 1:**
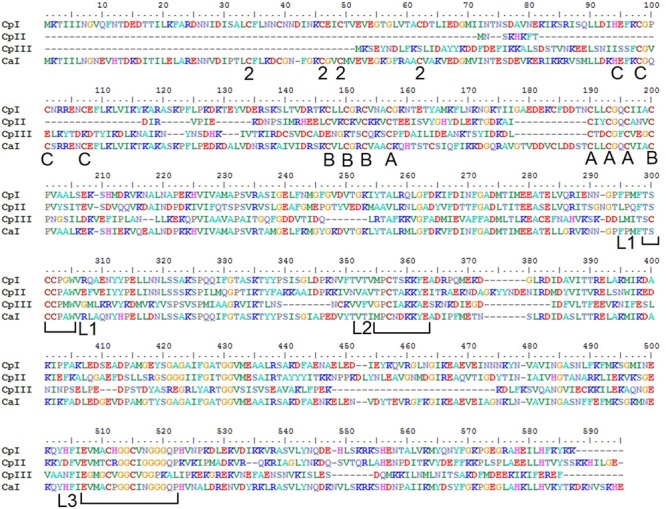
Protein sequence alignment of the [FeFe]-hydrogenases of *C. pasteurianum* W5 and *C. acetobutylicum*. Residues ligating FeS clusters are indicated with A, B, C, or 2 to denote the cluster they ligate; A – (FS4A), B – FS4B, C – FS4C, and 2 – FS2. The H-cluster coordinating motifs are designated by brackets showing L1, L2, and L3 (Vignais and Billoud, 2007).

**FIGURE 2:**
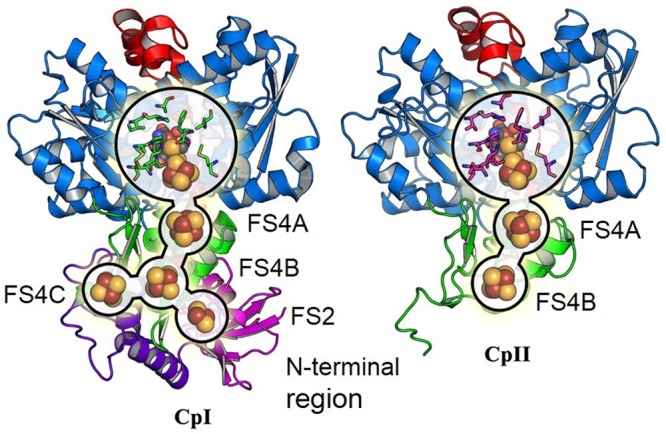
Comparison of overall protein structure of CpI and the CpII homology model.

The sequences of CpI and CpII are 33% identical, with 45% identity and 61% similarity over the conserved region (**Figure 1**), which suggests that these two enzymes have generally conserved protein architectures. A homology model of CpII (**Figure 2**) based on the solved crystal structure of CpI (Peters et al., 1998) and generated using SwissModel (Arnold et al., 2006), as well as amino acid sequence alignment, indicate the absence of accessory domains in CpII that are present in CpI. The CpI sequence contains conserved cysteine residues for each Fe/S cluster that sequentially bind clusters [2Fe-2S] (FS2), the distal [4Fe-4S] cluster (FS4C), the medial [4Fe-4S] cluster (FS4B) and the proximal [4Fe-4S] cluster (FS4A). In contrast, the N-terminus of CpII lacks the cysteine residues responsible for binding accessory Fe/S clusters FS2 and FS4C (**Figure 2**). However, conserved regions binding the two Fe atoms of the catalytic site, known as the H-cluster, and two [4Fe-4S] accessory clusters were identified in CpII. CpIII, which has thus far not been biochemically characterized, has a unique N-terminal arrangement of cysteines. Sequence alignment reveals that the FS4A binding motif is conserved, while the FS4B motif lacks two of the four cysteine residues that typically ligate this cluster (**Figure 1**). CpIII therefore has significant sequence differences from other biochemically characterized hydrogenases, which may provide it with intriguing properties.

The [FeFe]-hydrogenase sequences encoded in the CpW5 genome contain the evolutionarily conserved H-cluster motifs; TSCCPxW (L1), MPCxxKxxE (L2), and ExMxCxxGCxxG (L3) (**Figure 1**; Vignais and Billoud, 2007; Schmidt et al., 2010). These motifs include all of the H-cluster binding cysteines, as well as residues for ligating accessory clusters (**Figure 1**) Phylogenetic clustering of the H-cluster domains of clostridial [FeFe]-hydrogenases has shown a variety of distinct clusters, designated A1-A5, A7, A8, and B1-B3 (Calusinska et al., 2011). Group A2, which includes CpI, is comprised of monomeric, soluble, H_2_-producing enzymes. CpII, which lacks the [2Fe-2S] cluster as well as one of the [4Fe-4S] clusters, is classified as an A3 hydrogenase. Members of group B2, that includes CpIII, have an average size of 450 amino acids and an additional characteristic cysteine residue in the P1 motif (TS**C**CCPxW) of the H-cluster (Calusinska et al., 2010). No hydrogenases of this type have been biochemically characterized to date, and it is unclear if these sequences produce active hydrogenases. CpI, CpII, and CpIII are predicted to be monomeric, group 1 enzymes based on flanking gene analysis according to the classification system of Poudel et al., 2016, and are thus not expected to bifurcate (Poudel et al., 2016).

The [NiFe]-hydrogenase gene cluster of CpW5 contains the required accessory genes (*hypABCEFD* and *hoxN*) downstream of the structural genes, *hyaAB*, which encode the large and small subunits, respectively. The predicted protein sequence of the large subunit contains previously described (Vignais and Billoud, 2007) L1 and L2 motifs characteristic of membrane-bound, uptake hydrogenases. The L1 and L2 motifs encompass the highly conserved cysteine pairs (CxxC) near each terminus that ligate the NiFe center. Unlike [FeFe]-hydrogenases, maturases for the [NiFe]-hydrogenase are often found in a single gene cluster with the structural genes (Casalot and Rousset, 2001), as is the case for the CpW5 genome. This gene cluster is not co-localized with any other hydrogenase or nitrogenase genes. The gene for the hydrogenase large subunit (*hyaB*) clusters phylogenetically with other clostridia in group 1 (Calusinska et al., 2010) (data not shown), which comprises membrane-associated uptake hydrogenases (Vignais and Billoud, 2007). To understand hydrogenase metabolism in CpW5 fully, it was necessary to include transcriptional information for all of the encoded hydrogenases, including the [NiFe]-hydrogenase.

### Transcriptional and Physiological Analyses

Quantitative real-time PCR assays were performed to compare the transcript abundance of each CpW5 hydrogenase under both non-nitrogen-fixing and nitrogen-fixing conditions. Transcript levels for CpI decreased under nitrogen-fixing conditions (2.9-fold change) while CpII transcripts increased (7.5-fold change). The transcript levels of CpIII and the [NiFe]-hydrogenase were low with and without fixed nitrogen. For CpIII, relative transcript abundances were approximately 1% of those for CpI or CpII, based on the amplification threshold for each gene, and slightly lower under diazotrophic conditions (50% decrease). The [NiFe]-hydrogenase transcript levels were approximately 1% those of CpII under non-nitrogen-fixing conditions, but underwent an 8.7-fold increase during diazotrophic growth. Despite this increase in transcript abundance for the [NiFe]-hydrogenase, the relative transcript abundance of the [NiFe]-hydrogenase was approximately 10% of the CpII transcript abundance under nitrogen-fixing conditions. Thus, low levels of [NiFe]-hydrogenase transcripts are present under both nitrogen replete and nitrogen-fixing conditions.

Collectively, the abundance of hydrogenase transcripts agrees with previously established protein expression conditions and supports a rational model of hydrogenase usage by CpW5: CpI, which is known to be abundantly expressed under standard, non-nitrogen-fixing fermentative conditions (Adams et al., 1989), functions to dispose of excess reducing equivalents as H_2_, whereas under diazotrophic conditions, dinitrogen reduction by Mo-nitrogenase consumes a large amount of electrons and therefore subverts the need for an electron-consuming, proton-reducing enzyme. While CpI and CpII are ATP-independent, ATPases are necessary for nitrogenase to reduce dinitrogen to ammonia (Taylor, 1969).

**FIGURE 3:**
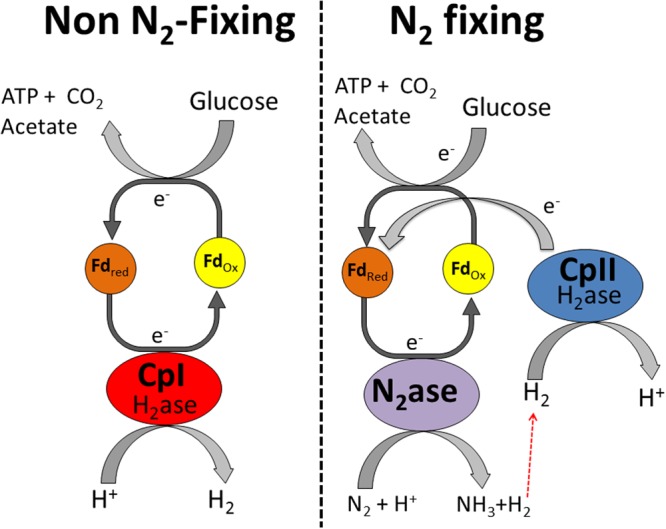
Proposed metabolic functions of CpI and CpII in *C. pasteurianum*. CpI acts as an electron sink under standard fermentative conditions, while CpII acts to recycle reducing equivalents in H_2_ during nitrogen fixation. It should be noted that the [NiFe]-hydrogenase may also function alongside CpII to recycle reducing equivalents, although this hydrogenase probably makes a smaller contribution than CpII to H_2_ oxidation.

In contrast, CpII has an exceptionally low H_2_ production activity (Adams and Mortenson, 1984b) and thus it is unlikely that it is capable of removing excess reducing equivalents. Rather, its high H_2_ oxidation activity and almost negligible proton reduction capacity is consistent with this hydrogenase functioning in the uptake direction. This H_2_ oxidation is of particular importance for recycling electrons from the nitrogenase-produced H_2_ and feeding those electrons back into the reductant-consuming, nitrogen-fixing metabolism. In this model, CpII thereby acts to recycle reducing equivalents, mitigating loss of electrons from H_2_ produced by nitrogenase (**Figure 3**). The specific catalytic abilities of CpI and CpII, along with the changes in transcript abundance, suggest that there is little interaction between these hydrogenases. The [NiFe]-hydrogenase has a similar transcriptional profile to CpII, which is up-regulated under nitrogen-fixing conditions. This suggests that the [NiFe]-hydrogenase may also have a role in recapturing reducing equivalents, as has been previously demonstrated for [NiFe]-hydrogenases in aerobic, nitrogen-fixing organisms (Walker and Yates, 1978; Laane et al., 1979; Walker et al., 1981; Hamilton et al., 2011), but our data suggest a much lower abundance of [NiFe]-hydrogenase transcripts compared to CpII transcripts. *In vitro* data has shown that *C. pasteurianum* hydrogenase donates electrons to nitrogenase (Mortenson, 1964), similar to the observed roles of hydrogenases across a diversity of species (Robson and Postgate, 1980; Bothe et al., 2010). The qRT-PCR data are consistent with previous observations that CpII is expressed primarily under nitrogen-fixing conditions and that CpI and CpII account for the majority of the total hydrogenase activity observed during protein purification (Chen and Blanchard, 1978; Adams and Mortenson, 1984b). The proficiency of CpII at H_2_ oxidation precludes the necessity of the [NiFe]-hydrogenase functioning to recycle reducing equivalents during nitrogen fixation, however, it may have its own metabolic niche, such as in Fe-limited circumstances. In the case of CpIII, the miniscule changes in transcript abundance, and apparent lack of protein expression, suggest that this enzyme does not significantly contribute to *C. pasteurianum* metabolism under conditions such as those measured in this study.

The different catalytic rates and metabolic functions of CpI and CpII provide a unique system for the study of catalytic bias. The specific catalytic abilities of these hydrogenases function to enhance one direction of the reaction while minimizing the other; they demonstrate the complex interplay between the metabolic niche of a protein and the structural fine-tuning it must possess to perform a particular function. There is a large selective advantage to having an enzyme with very low rates of proton reduction, such as CpII, because such enzymes are likely to be operating near equilibrium under nitrogen-fixing conditions, and the slow rates of proton reduction would reduce the loss of precious reducing equivalents.

Of more than 40 fully sequenced genomes of *Clostridium* species, all but two (*C. kluyveri* and *C. butyricum*) encode nitrogenase and a [NiFe]-hydrogenase. The two exceptions have genes encoding three or more [FeFe]-hydrogenases, suggesting that one or more of the [FeFe]-hydrogenases serves to consume H_2_ and thereby supplants the need for a [NiFe]-hydrogenase during nitrogen fixation.

### Structural Basis for Catalytic Bias in [FeFe]-Hydrogenases

[FeFe]-hydrogenase 1 from CpW5 and CpII display substantial differences in the ability to reduce protons and oxidize H_2_, and we hypothesize that these differences are due to structural differences and thus to their amino acid sequences. Comparison of activities among hydrogenases does not reveal a trend in H_2_ oxidation to production ratios based on the differing FeS cluster binding motifs (Mulder et al., 2011). It is currently unclear to what extent the presence of additional Fe/S clusters contributes to differences in catalytic biases amongst these hydrogenases. Most likely, a suite of structural features is responsible for tuning the directionality of a given hydrogenase. The particular amino acids involved in gas channel lining (Liebgott et al., 2011), proton transfer (Morra et al., 2015), electron transfer, and H-cluster ligand environment (Knörzer et al., 2012) may all play a role.

**FIGURE 4:**
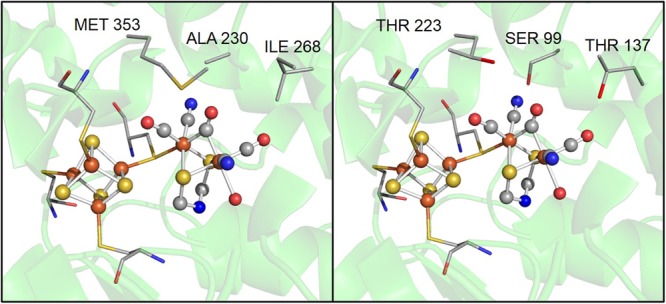
A zoomed in comparison of several possible key residues in the active site that influence catalytic bias, including, alanine 230, isoleucine 268, and methionine 353 in CpI, which correspond to a serine, threonine, and threonine in CpII, respectively.

Although the motifs coordinating H-clusters are conserved across [FeFe]-hydrogenases, amino acids in the second coordination sphere are not conserved. For example, three residues near the 2Fe subcluster, A230, I268, and M353 in CpI, are S99, T137, and T223 in CpII (**Figure 4**). These differences highlight how variation in the second coordination sphere may play a role in modulating catalytic bias. Previous work by Knörzer et al. (2012) showed that Thr (T137 in CpII) is the most frequent substitution for a Met (residue 353 in CpI) that is adjacent to the μ-CO of the 2Fe subcluster, in 409 CpI homologs. These authors used site-directed substitution to change M353 to L353 in CpI and observed a significant decrease in H_2_ production (to 15% of WT enzyme) and a small decrease in H_2_ oxidation (to 74% of WT), which they attributed to a lower turnover rate (Knörzer et al., 2012). This suggests that close proximity of this residue to 2Fe influences the enzymatic preference for H_2_ oxidation or production, and that Leu results in an enzyme that favors oxidation to a greater degree relative to one that has Met.

**FIGURE 5:**
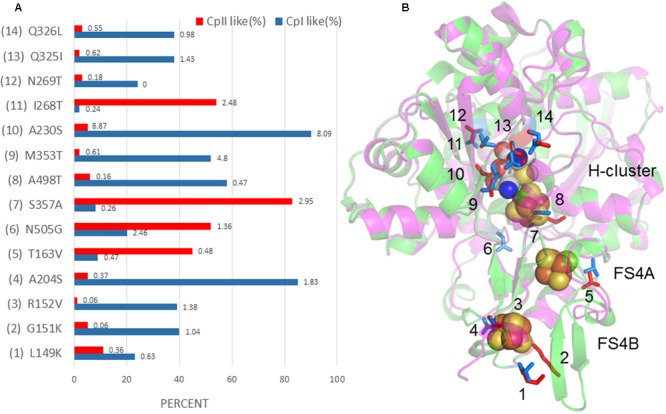
**(A)** Percent conservation of either CpI- or CpII-type residues among 829 [FeFe]-hydrogenase sequences is displayed in blue and red, respectively, along the *X*-axis. The *Y*-axis displays sites corresponding to the model depicted in **(B)**, where CpII is superimposed with CpI, and 14 sites have been identified that may tune the FeS cluster potential. Residues are numbered according to CpI. The number at the end of each bar in **(A)** is the *K*-value, or phylogenetic signal, that corresponds to the amino acid at that position. A value of 1 or greater shows a high degree of phylogenetic signal, or conservation, and can be interpreted to indicate that this residue is under strong selective pressure.

A further comparison of the crystal structure of CpI with the CpII homology model revealed 14 potential sites (four at the FS4B, one at the FS4A, and nine in the H-cluster region) that may influence the redox potential of the Fe/S centers and thereby alter the catalytic bias (**Figure 5**). These sites were identified by examining amino acids that differed between CpI and CpII and were found within 5 Å of the FeS clusters. By cross-checking these residues with their conservation percentage and phylogenetic signal (K-statistic) among [FeFe]-hydrogenases, it is evident that most of these 14 residues are under strong selective pressure, demonstrating the functional importance of these residues and positions. These findings agree with evidence suggesting the importance of accessory clusters on the catalytic properties of hydrogenases (Abou Hamdan et al., 2012; Winkler et al., 2013). Electrochemical investigations provide evidence that the terminal cluster of the electron transfer pathway within enzymes influence catalytic bias (Hexter et al., 2012), for example, in *E. coli* [NiFe]-hydrogenase 1 (Armstrong et al., 2016). Most likely, the determinants of catalytic bias are not found at a single site, but are rather a suite of residues that act in concert with one another. The work presented here suggests 14 specific amino acids that may influence the electronic properties of the accessory FeS clusters as well as the active site, and provides a platform for future studies using a site-directed mutagenesis approach.

## Conclusion

In this work the complement of hydrogenases from CpW5 was analyzed to provide better insights into the H_2_ metabolism of this organism. The genome revealed sequences of three [FeFe]-hydrogenases and a [NiFe]-hydrogenase that allowed, for the first time, a comparison of the deduced amino acid sequences of the two biochemically characterized [FeFe]-hydrogenases, CpI and CpII, which have a sequence similarity of 61%. Targeted transcriptional analyses are consistent with a role for CpII in recapturing the reducing equivalents that are produced as H_2_ as part of Mo-nitrogenase catalysis during growth under nitrogen-fixing conditions. A role for CpII in H_2_ uptake is also consistent with the previously observed catalytic bias of CpII toward H_2_ oxidation. CpII probably evolved to be a poor proton-reducing enzyme, thereby limiting potential loss of H_2_ under nitrogen-fixing conditions when the availability of reducing equivalents may be growth-limiting. A comparison of the CpI and CpII sequences in the context of their respective phylogenetic and structural relationships reveal several likely determinants of catalytic bias, which can be studied by characterization of site-specific variants of these [FeFe]-hydrogenases.

## Materials and Methods

### Growth Conditions

Freeze-dried *C. pasteurianum* strain W5 (ATCC^®^ 6013^TM^) was obtained from ATCC and rehydrated with Difco^®^ Reinforced Clostridial (DRC) Medium following the ATCC protocol. Sealed 25 ml glass serum vials (Wheaton) containing 10 ml of DRC medium under a headspace of 10% H_2_-10% CO_2_-80% N_2_ were then inoculated with the rehydrated culture and incubated at 37°C following ATCC propagation procedures for this organism. Agar plates prepared with DRC medium were used to store *C. pasteurianum* strain W5 for further use. Plates were incubated at 37°C for 24–48 h and were then stored at room temperature in an anaerobic chamber.

For genome sequencing, cultures were inoculated from a single colony from a DRC agar plate that was inoculated into a sealed 25-ml serum vials containing 10 ml of the same medium. Cells were grown under a headspace of 10% H_2_-10% CO_2_-80% N_2_ by overnight incubation at 37°C. An aliquot (1 ml) of the culture was spun down at 14,000 × *g* at room temperature before extracting DNA.

### Genome Sequencing

Total genomic DNA of *C. pasteurianum* strain W5 was extracted using a Promega Wizard^®^ Plus SV minipreps DNA purification system. The concentration (220 ng/μL) was determined by a NanoDrop 1000 Spectrophotometer (Abs_260/280_ = 2.04). Genomic DNA was submitted to the Genomics Core Facility at The Pennsylvania State University for 454 pyrosequencing (Mardis, 2008). Reads were assembled with the Newbler assembler (ver. 2.6; Roche) into 145 contigs of at least 500 bp with 116 of those contigs predicted to form six large scaffolds. The read depth was about 19X.

Gaps were closed by PCR using primers designed approximately 200 bases from the end of each contig. GoTaq^®^ 2x Master Mix (Promega, Madison, WI, United States) was used for the amplification reactions in a Techne Touchgene Gradient Thermal Cycler (Techne, Bulington, NJ, United States). Amplicons were then purified either directly using QIAquick PCR Purification Kit or from agarose gels using the Qiaex II Gel Extraction Kit (Qiagen, Valencia, CA, United States). Purified PCR products were sequenced by Davis Sequencing in Davis, CA, and sequence data were assembled using BioEdit (v7). The final assembled reads were compared to the recently published closed genome [9] using the ANI calculator tool with default settings (Rodriguez-R and Konstantinidis, 2016) using previously described methods (Goris et al., 2007; Rodriguez-R and Konstantinidis, 2014). This Whole Genome Shotgun project has been deposited at DDBJ/EMBL/GenBank under the accession LFYL00000000. The version described here is version LFYL01000000.

The genome was annotated using the RAST (Rapid Annotation using Subsystem Technology) genome annotation server (Overbeek et al., 2014). The amino acid sequence of the H-cluster domain of CpI was used as a BLAST query against a database of the amino acid sequences encoded by the draft genome. All matches with an expect value (*e*-value) below 1.0 were aligned using ClustalW to determine whether they contained previously published signature motifs L1, L2, and L3 found in [FeFe]-hydrogenases.

For qRT-PCR, cultures were inoculated from a single colony from a DRC agar plate into a sealed 25-ml serum vial containing 10 ml of the same medium. Cells were grown under a headspace of 10% H_2_-10% CO_2_-80% N_2_ by overnight incubation at 37°C. One to five ml of overnight culture was used to inoculate 50 ml of both DM-11 (Mallette et al., 1974) (N+; containing NH_4_Cl and (NH_4_)_2_SO_4_) and DM-11-N (N–; fixed nitrogen free) media to an OD_650_ = 0.020 and were then sparged with 10% H_2_-10% CO_2_-80% N_2_ for 10 min and incubated overnight. This was repeated after which point a C_2_H_2_ reduction assay (Hardy et al., 1968) was performed to ensure that the N- culture contained nitrogenase activity. In sealed 120-mL serum vials, 1 mL (N+) cell culture was added to 50 mL of N+ media, and 6 mL of (N–) cell culture were added to 50 mL of N– media, to give an initial OD_650_ = 0.020 for each culture. The vials were once again sparged with the gas mix and incubated overnight at 37°C. The following day, another C_2_H_2_ reduction assay was performed to verify nitrogenase activity. Samples (500 μl) were mixed with 1 ml of RNAprotect Bacterial Reagent^®^ (Qiagen, Valencia, CA, United States) and either subjected to RNA extraction immediately or frozen at –20°C until later RNA extraction.

### Preparation of Total RNA

Total RNA was extracted from *C. pasteurianum* strain W5 using a RNeasy^®^ Mini Kit (Qiagen, Valencia, CA, United States) according to the manufacturer’s protocol. For N+ conditions, the OD_650_ of the cell culture was 0.9, while for N– cultures it was 0.4. Note that N– cultures reach stationary phase at a density of nearly half the N+ cultures. In both cases, cells were in the exponential phase at the time of harvesting. The DNase treatment step used the RQ1 RNase-Free DNase (Promega, Madison, WI, United States) and a re-purification using the RNeasy^®^ Mini Kit. The RNA concentration was determined using Qubit^®^ RNA Assay Kit (Life Technologies, Carlsbad, CA, United States), and the RNA solution was stored at –20 °C until further analysis.

### Quantitative RT-PCR (qRT-PCR)

Integrated DNA Technologies SciTools qPCR online primer designing software was used to design primers for the four hydrogenases (CpI, CpII, CpIII and the large subunit of the [NiFe]-hydrogenase, Supplementary Table 1). The nitrogenase α-subunit, *nifD*, and the 16S rRNA small subunit (Supplementary Table 1), served as controls, to which expression was normalized. qRT-PCR was performed on a Rotor-Gene-Q real-time PCR detection system (Qiagen, Valencia, CA, United States) using the *Power* SYBR^®^ green RNA-to-$C_{T}^{\mathrm{TM}}$ *1-Step* Kit (Life Technologies, Carlsbad, CA, United States) according to the specified protocol. Briefly, each reaction consisted of 10 μL *Power* SYBR^®^ Green PCR master mix, 100 nM each of the forward and reverse primers, 100 ng of RNA template, and nuclease-free H_2_O to a final volume of 20 μL. Cycling conditions were 40 min at 48°C, 10 min at 95°C, and then 40 cycles of 15 s at 95°C, and then 1 min at 60°C. Reactions were performed in triplicate with control reaction mixtures containing no reverse transcriptase. Each transcriptional experiment was repeated a minimum of three times using RNA isolated from separate cultures. Primer efficiencies for DNA were 0.98 for 16S rRNA gene, 0.97 for the CpI gene, 0.92 for the CpII gene, 0.84 for the CpIII gene, and 0.88 for *nifD*, using a DNA concentration of 145 ng μl^-1^ to 0.145 ng μl^-1^.

### Bioinformatics

Homologs of CpI were compiled from the Integrated Microbial Genomes (IMG) database (Markowitz et al., 2012) using BLASTp, resulting in 829 protein sequences. CpI and CpII, along with 829 homologs, were subjected to a multiple sequence alignment (MSA) using the Muscle algorithm as implemented in MEGA (vers. 6) (Tamura et al., 2013) with default settings. Residues at each aligned position were removed if they were found to be identical in both CpI and CpII. For each of the remaining residues the degree of conservation (as a percent) among the 829 homologs was calculated using the MSA. SWISS-MODEL (Schwede et al., 2003) was used to generate a homology model of CpII, based upon the structure of CpI (Peters et al., 1998). Pymol (Delano, 2002) was then used to superimpose CpII onto CpI with a structure-based alignment. Based on this superimposition, functionally important residues that differed between CpI and CpII were selected. Sites examined included the proton transfer channel (Cornish et al., 2011) and the protein sphere around the H-cluster, as well as the proximal and medial [4Fe-4S] clusters. Using this set of criteria, a total of 14 residues were identified in the Fe/S cluster regions that potentially differentiate the functionality of these enzymes.

The large subunit of [FeFe]-hydrogenase (HydA) contains an H-cluster domain containing at least ∼350 residues (Meyer, 2007; Vignais and Billoud, 2007). In addition to the H cluster, *hydA* often encodes diverse N-terminal (F-cluster) and C-terminal (C-cluster) domains. To minimize bias in phylogenetic reconstruction, the alignment containing the 829 homologous hydrogenases, as well as CpI and CpII, was trimmed to contain just the H-cluster domain, as previously described (Mulder et al., 2010). A phylogenetic tree of the H-cluster alignment block was constructed using a maximum likelihood method, i.e., RaxML, specifying the following parameters: gamma rate distribution, fixed base frequencies, and the BLOSUM62 substitution matrix (Stamatakis, 2014). The tree was rooted with Nar1 proteins from *Homo sapiens* (NP_036468, NP_071938) and *Danio rerio* (A2RRV9). The phylogenetic signal (K-statistic) associated with the distribution of the 14 individual amino acids at each of the identified alignment positions, as they are distributed on the H-cluster phylogenetic tree, was quantified using the program multiphylosignal within the Picante package (Kembel, 2010) as implemented with the base package R. The K statistic compares the observed signal in the distribution of a trait (e.g., particular amino acid usage at a specific alignment position) on a phylogeny to the signal under a Brownian motion model of evolution (Blomberg et al., 2003). Values of *K* that are close to 1 imply a Brownian motion for the evolution of that trait (or some degree of phylogenetic signal) while values greater than 1 indicate strong phylogenetic signal. *K* values closer to zero or which are negative correspond to a random or convergent pattern of evolution for that trait.

Based on the H-cluster phylogenetic tree constructed above, 39 hydrogenase homologs that grouped phylogenetically with CpI and 39 homologous hydrogenases that grouped with CpII were identified. These 78 hydrogenases along with CpI and CpII were aligned and subjected to phylogenetic reconstruction as described above. The F- and C-cluster domains of the hydrogenases were identified using BLASTp against the Conserved Domain Database (CDD) and the CDSEARCH/cdd v3.13 algorithm (Marchler-Bauer et al., 2015) (version 3.13) using an *e*-value of 0.01 as previously described (Calusinska et al., 2010). These CpI- and CpII-like hydrogenases were categorized into modular structures based on the presence of identified F- and C- clusters as described previously (Calusinska et al., 2010). The modular structure was then overlaid onto the respective tree to determine the extent to which phylogeny predicts the distribution of F- and C-clusters. The genomes of organisms with the previously identified 78 hydrogenase homologs were screened for NifH using BLASTp and the NifH sequence from Cp as a search query. The distribution of *nifH* in the genomes of the respective taxa was also mapped onto the respective phylogenetic trees (Supplementary Figures 2, 3). Interactive Tree Of Life (iTOL) was used to project the phylogenetic trees (Letunic and Bork, 2007).

## Author Contributions

JT performed Clostridial growth and qRT-PCR experiments, JA and SP carried out bioinformatics studies. JT and JA wrote the document. EB, TH, and JP contributed to experimental design and manuscript revision. ZL, SN, and DB sequenced the genome. PK and MA contributed to data interpretation. All authors read and approved the final manuscript.

## Conflict of Interest Statement

The authors declare that the research was conducted in the absence of any commercial or financial relationships that could be construed as a potential conflict of interest.

## Abbreviations

CpI: [FeFe]-hydrogenase 1 from CpW5
CpII: [FeFe]-hydrogenase 2 from CpW5
CpIII: [FeFe]-hydrogenase 3 from CpW5
CpW5: *Clostridium pasteurianum* ATCC 6013 (strain W5)
WT: wild type

## References

[B1] Abou Hamdan A. Dementin S. Liebgott P.-P. Gutierrez-Sanz O. Richaud P. De Lacey A. L. (2012). Understanding and tuning the catalytic bias of hydrogenase. *J. Am. Chem. Soc.* 134 8368–8371. 10.1021/ja301802r 22540997

[B2] Adams M. W. (1990). The structure and mechanism of iron-hydrogenases. *Biochim. Biophys. Acta* 1020 115–145. 10.1016/0005-2728(90)90044-5 2173950

[B3] Adams M. W. Eccleston E. Howard J. B. (1989). Iron-sulfur clusters of hydrogenase I and hydrogenase II of *Clostridium pasteurianum*. *Proc. Natl. Acad. Sci. U.S.A.* 86 4932–4936. 10.1073/pnas.86.13.4932 2544883 PMC297529

[B4] Adams M. W. W. Mortenson L. E. (1984a). The physical and catalytic properties of hydrogenase II of *Clostridium pasteurianum*. A comparison with hydrogenase I. *J. Biol. Chem.* 259 7045–7055. 6327705

[B5] Adams M. W. W. Mortenson L. E. (1984b). The purification of hydrogenase II (uptake hydrogenase) from the anaerobic N2-fixing bacterium *Clostridium pasteurianum*. *Biochim. Biophys. Acta* 766 51–61. 10.1016/0005-2728(84)90216-0

[B6] Armstrong F. A. Evans R. M. Hexter S. V. Murphy B. J. Roessler M. M. Wulff P. (2016). Guiding principles of hydrogenase catalysis instigated and clarified by protein film electrochemistry. *Acc. Chem. Res.* 49 884–892. 10.1021/acs.accounts.6b00027 27104487

[B7] Arnold K. Bordoli L. Kopp J. Schwede T. (2006). The SWISS-MODEL workspace: a web-based environment for protein structure homology modelling. *Bioinformatics* 22 195–201. 10.1093/bioinformatics/bti770 16301204

[B8] Baltazar C. S. A. Marques M. C. Soares C. M. DeLacey A. M. Pereira I. A. C. Matias P. M. (2011). Nickel–iron–selenium hydrogenases – an overview. *Eur. J. Inorg. Chem.* 2011 948–962. 10.1002/ejic.201001127

[B9] Bettegowda C. Huang X. Lin J. Cheong I. Kohli M. Szabo S. A. (2006). The genome and transcriptomes of the anti-tumor agent *Clostridium novyi-NT*. *Nat. Biotechnol.* 24 1573–1580. 10.1038/nbt1256 17115055 PMC9338427

[B10] Blomberg S. P. Garland J. T. Ives A. R. (2003). Testing for phylogenetic signal in comparative data: behavioral traits are more labile. *Evolution* 57 717–745. 10.1111/j.0014-3820.2003.tb00285.x 12778543

[B11] Bothe H. Schmitz O. Yates M. G. Newton W. E. (2010). Nitrogen fixation and hydrogen metabolism in cyanobacteria. *Microbiol. Mol. Biol. Rev.* 74 529–551. 10.1128/mmbr.00033-10 21119016 PMC3008169

[B12] Brecht M. van Gastel M. Buhrke T. Friedrich B. Lubitz W. (2003). Direct detection of a hydrogen ligand in the [NiFe] center of the regulatory H2-sensing hydrogenase from *Ralstonia eutropha* in its reduced state by HYSCORE and ENDOR spectroscopy. *J. Am. Chem. Soc.* 125 13075–13083. 10.1021/ja036624x 14570480

[B13] Calusinska M. Happe T. Joris B. Wilmotte A. (2010). The surprising diversity of clostridial hydrogenases: a comparative genomic perspective. *Microbiology* 156(Pt 6) 1575–1588. 10.1099/mic.0.032771-0 20395274

[B14] Calusinska M. Joris B. Wilmotte A. (2011). Genetic diversity and amplification of different clostridial [FeFe] hydrogenases by group-specific degenerate primers. *Lett. Appl. Microbiol.* 53 473–480. 10.1111/j.1472-765X.2011.03135.x 21838748

[B15] Casalot L. Rousset M. (2001). Maturation of the [NiFe] hydrogenases. *Trends Microbiol.* 9 228–237. 10.1016/S0966-842X(01)02009-1 11336840

[B16] Chen J.-S. Blanchard D. K. (1978). Isolation and properties of a unidirectional H2-oxidizing hydrogenase from the strictly anaerobic N2-fixing bacterium *Clostridium pasteurianum* W5. *Biochem. Biophys. Res. Commun.* 84 1144–1150. 10.1016/0006-291X(78)91703-5 728150

[B17] Cornish A. J. Gartner K. Yang H. Peters J. W. Hegg E. L. (2011). Mechanism of proton transfer in [FeFe]-hydrogenase from *Clostridium pasteurianum*. *J. Biol. Chem.* 286 38341–38347. 10.1074/jbc.M111.254664 21900241 PMC3207428

[B18] Delano W. L. (2002). *The PyMOL Molecular Graphics System.* Available at: http://www.pymol.org

[B19] Dementin S. Burlat B. De Lacey A. L. Pardo A. Adryanczyk-Perrier G. Guigliarelli B. (2004). A glutamate is the essential proton transfer gate during the catalytic cycle of the [NiFe] hydrogenase. *J. Biol. Chem.* 279 10508–10513. 10.1074/jbc.M312716200 14688251

[B20] Ghirardi M. L. Cohen J. King P. Schulten K. Kim K. Seibert M. (2006). “[FeFe]-hydrogenases and photobiological hydrogen production,” in *Proceedings of the SPIE 6340 Solar Hydrogen and Nanotechnology, 63400X* San Diego, CA.

[B21] Goris J. Konstantinidis K. T. Klappenbach J. A. Coenye T. Vandamme P. Tiedje J. M. (2007). DNA-DNA hybridization values and their relationship to whole-genome sequence similarities. *Int. J. Syst. Evol. Microbiol.* 57(Pt 1) 81–91. 10.1099/ijs.0.64483-0 17220447

[B22] Hamilton T. L. Ludwig M. Dixon R. Boyd E. S. Dos Santos P. C. Setubal J. C. (2011). Transcriptional profiling of nitrogen fixation in *Azotobacter vinelandii*. *J. Bacteriol.* 193 4477–4486. 10.1128/jb.05099-11 21724999 PMC3165507

[B23] Hardy R. W. F. Holsten R. D. Jackson E. K. Burns R. C. (1968). The acetylene-ethylene assay for N_2_ fixation: laboratory and field evaluation. *Plant Physiol.* 43 1185–1207. 10.1104/pp.43.8.1185 16656902 PMC1086994

[B24] Hedderich R. (2004). Energy-converting [NiFe] hydrogenases from archaea and extremophiles: ancestors of complex I. *J. Bioenerg. Biomembr.* 36 65–75. 10.1023/b:jobb.0000019599.43969.33 15168611

[B25] Hexter S. V. Grey F. Happe T. Climent V. Armstrong F. A. (2012). Electrocatalytic mechanism of reversible hydrogen cycling by enzymes and distinctions between the major classes of hydrogenases. *Proc. Natl. Acad. Sci. U.S.A.* 109 11516–11521. 10.1073/pnas.1204770109 22802675 PMC3406873

[B26] Kanai T. Matsuoka R. Beppu H. Nakajima A. Okada Y. Atomi H. (2011). Distinct physiological roles of the three [NiFe]-hydrogenase orthologs in the hyperthermophilic archaeon *Thermococcus kodakarensis*. *J. Bacteriol.* 193 3109–3116. 10.1128/jb.01072-10 21515783 PMC3133214

[B27] Kembel S. (2010). *An Introduction to the Picante Package.*

[B28] Knörzer P. Silakov A. Foster C. E. Armstrong F. A. Lubitz W. Happe T. (2012). Importance of the protein framework for catalytic activity of [FeFe]-hydrogenases. *J. Biol. Chem.* 287 1489–1499. 10.1074/jbc.M111.305797 22110126 PMC3256906

[B29] Kolek J. Sedlář K. Provazník I. Patáková P. (2014). Draft genome sequence of *Clostridium pasteurianum* NRRL B-598 a potential butanol or hydrogen producer. *Genome Announc.* 2: e00192-14 10.1128/genomeA.00192-14 PMC3961727 24652980

[B30] Laane C. Haaker H. Veeger C. (1979). On the efficiency of oxidative phosphorylation in membrane vesicles of *Azotobacter vinelandii* and of *Rhizobium leguminosarum* Bacteroids. *Eur. J. Biochem.* 97 369–377. 10.1111/j.1432-1033.1979.tb13123.x 223842

[B31] Lenz O. Bernhard M. Buhrke T. Schwartz E. Friedrich B. (2002). The hydrogen-sensing apparatus in *Ralstonia eutropha*. *J. Mol. Microbiol. Biotechnol.* 4 255–262. 11931556

[B32] Letunic I. Bork P. (2007). Interactive Tree Of Life (iTOL): an online tool for phylogenetic tree display and annotation. *Bioinformatics* 23 127–128. 10.1093/bioinformatics/btl529 17050570

[B33] Liebgott P.-P. de Lacey A. L. Burlat B. Cournac L. Richaud P. Brugna M. (2011). Original design of an oxygen-tolerant [NiFe] hydrogenase: major effect of a valine-to-cysteine mutation near the active site. *J. Am. Chem. Soc.* 133 986–997. 10.1021/ja108787s 21175174

[B34] Mallette M. F. Reece P. Dawes E. A. (1974). Culture of *Clostridium pasteurianum* in defined medium and growth as a function of sulfate concentration. *Appl. Microbiol.* 28 999–1003. 4451381 10.1128/am.28.6.999-1003.1974 PMC186871

[B35] Marchler-Bauer A. Derbyshire M. K. Gonzales N. R. Lu S. Chitsaz F. Geer L. Y. (2015). CDD: NCBI’s conserved domain database. *Nucleic Acids Res.* 43 D222–D226. 10.1093/nar/gku1221 25414356 PMC4383992

[B36] Mardis E. R. (2008). Next-generation DNA sequencing methods. *Annu. Rev. Genomics Hum. Genet.* 9 387–402. 10.1146/annurev.genom.9.081307.164359 18576944

[B37] Markowitz V. M. Chen I.-M. A. Palaniappan K. Chu K. Szeto E. Grechkin Y. (2012). IMG: the integrated microbial genomes database and comparative analysis system. *Nucleic Acids Res.* 40 D115–D122. 10.1093/nar/gkr1044 22194640 PMC3245086

[B38] Melis A. Seibert M. Happe T. (2004). Genomics of green algal hydrogen research. *Photosynth. Res.* 82 277–288. 10.1007/s11120-004-2050-2 16143840

[B39] Meyer J. (2007). [FeFe] hydrogenases and their evolution: a genomic perspective. *Cell. Mol. Life Sci.* 64 1063–1084. 10.1007/s00018-007-6477-4 17353991 PMC11136429

[B40] Morra S. Maurelli S. Chiesa M. Mulder D. W. Ratzloff M. W. Giamello E. (2015). The effect of a C298D mutation in CaHydA [FeFe]-hydrogenase: insights into the protein-metal cluster interaction by EPR and FTIR spectroscopic investigation. *Biochim. Biophys. Acta* 1857 98–106. 10.1016/j.bbabio.2015.10.005 26482707

[B41] Mortenson L. E. (1964). Ferredoxin and ATP, requirements for nitrogen fixation in cell-free extracts of *Clostridium pasteurianum*. *Proc. Natl. Acad. Sci. U.S.A.* 52 272–279. 10.1073/pnas.52.2.272 14206590 PMC300271

[B42] Mulder D. W. Boyd E. S. Sarma R. Lange R. K. Endrizzi J. A. Broderick J. B. (2010). Stepwise [FeFe]-hydrogenase H-cluster assembly revealed in the structure of HydAΔEFG. *Nature* 465 248–251. 10.1038/nature08993 20418861

[B43] Mulder D. W. Shepard E. M. Meuser J. E. Joshi N. King P. W. Posewitz M. C. (2011). Insights into FeFe -hydrogenase structure, mechanism, and maturation. *Structure* 19 1038–1052. 10.1016/j.str.2011.06.008 21827941

[B44] Nolling J. Breton G. Omelchenko M. V. Makarova K. S. Zeng Q. Gibson R. (2001). Genome sequence and comparative analysis of the solvent-producing bacterium *Clostridium acetobutylicum*. *J. Bacteriol.* 183 4823–4838. 10.1128/jb.183.16.4823-4838.2001 11466286 PMC99537

[B45] Overbeek R. Olson R. Pusch G. D. Olsen G. J. Davis J. J. Disz T. (2014). The SEED and the rapid annotation of microbial genomes using subsystems technology (RAST). *Nucleic Acids Res.* 42 D206–D214. 10.1093/nar/gkt1226 24293654 PMC3965101

[B46] Patakova P. Linhova M. Rychtera M. Paulova L. Melzoch K. (2013). Novel and neglected issues of acetone-butanol-ethanol (ABE) fermentation by clostridia: *Clostridium* metabolic diversity, tools for process mapping and continuous fermentation systems. *Biotechnol. Adv.* 31 58–67. 10.1016/j.biotechadv.2012.01.010 22306328

[B47] Peters J. W. Lanzilotta W. N. Lemon B. J. Seefeldt L. C. (1998). X-ray crystal structure of the Fe-only hydrogenase (CpI) from *Clostridium pasteurianum* to 1.8 angstrom resolution. *Science* 282 1853–1858. 10.1126/science.282.5395.1853 9836629

[B48] Poehlein A. Riegel K. Konig S. M. Leimbach A. Daniel R. Durre P. (2015). Genome sequence of *Clostridium sporogenes* DSM 795(T), an amino acid-degrading, nontoxic surrogate of neurotoxin-producing *Clostridium botulinum*. *Stand. Genomic Sci.* 10:40 10.1186/s40793-015-0016-y PMC4517662 26221421

[B49] Poudel S. Tokmina-Lukaszewska M. Colman D. R. Refai M. Schut G. J. King P. W. (2016). Unification of [FeFe]-hydrogenases into three structural and functional groups. *Biochim. Biophys. Acta* 1860 1910–1921. 10.1016/j.bbagen.2016.05.034 27241847

[B50] Pyne M. E. Utturkar S. Brown S. D. Moo-Young M. Chung D. A. Chou C. P. (2014). Improved draft genome sequence of *Clostridium pasteurianum* strain ATCC 6013 (DSM 525) using a hybrid next-generation sequencing approach. *Genome Announc.* 2:e00790-14 10.1128/genomeA.00790-14 PMC4125779 25103768

[B51] Robson R. L. Postgate J. R. (1980). Oxygen and hydrogen in biological nitrogen fixation. *Annu. Rev. Microbiol.* 34 183–207. 10.1146/annurev.mi.34.100180.001151 6776883

[B52] Rodriguez-R L. Konstantinidis K. (2014). Bypassing cultivation to identify bacterial species. *Microbe* 9 111–118. 10.1128/microbe.9.111.1

[B53] Rodriguez-R L. M. Konstantinidis K. T. (2016). The enveomics collection: a toolbox for specialized analyses of microbial genomes and metagenomes. *PeerJ Prepr.* 4:e1900v1 10.7287/peerj.preprints.1900v1

[B54] Rotta C. Poehlein A. Schwarz K. McClure P. Daniel R. Minton N. P. (2015). Closed genome sequence of *Clostridium pasteurianum* ATCC 6013. *Genome Announc.* 3:e01596-14. 10.1128/genomeA.01596-14 PMC4335343 25700419

[B55] Sakaguchi Y. Hayashi T. Kurokawa K. Nakayama K. Oshima K. Fujinaga Y. (2005). The genome sequence of *Clostridium botulinum* type C neurotoxin-converting phage and the molecular mechanisms of unstable lysogeny. *Proc. Natl. Acad. Sci. U.S.A.* 102 17472–17477. 10.1073/pnas.0505503102 16287978 PMC1283531

[B56] Schmidt O. Drake H. L. Horn M. A. (2010). Hitherto unknown [Fe-Fe]-hydrogenase gene diversity in anaerobes and anoxic enrichments from a moderately acidic fen. *Appl. Environ. Microbiol.* 76 2027–2031. 10.1128/aem.02895-09 20118375 PMC2838027

[B57] Schut G. J. Adams M. W. W. (2009). The iron-hydrogenase of *Thermotoga maritima* utilizes ferredoxin and NADH synergistically: a new perspective on anaerobic hydrogen production. *J. Bacteriol.* 191 4451–4457. 10.1128/jb.01582-08 19411328 PMC2698477

[B58] Schwede T. Kopp J. Guex N. Peitsch M. C. (2003). SWISS-MODEL: an automated protein homology-modeling server. *Nucleic Acids Res.* 31 3381–3385. 10.1093/nar/gkg520 12824332 PMC168927

[B59] Sebaihia M. Peck M. W. Minton N. P. Thomson N. R. Holden M. T. Mitchell W. J. (2007). Genome sequence of a proteolytic (Group I) *Clostridium botulinum* strain hall A and comparative analysis of the clostridial genomes. *Genome Res.* 17 1082–1092. 10.1101/gr.6282807 17519437 PMC1899119

[B60] Sedlar K. Kolek J. Skutkova H. Branska B. Provaznik I. Patakova P. (2015). Complete genome sequence of *Clostridium pasteurianum* NRRL B-598 a non-type strain producing butanol. *J. Biotechnol.* 214 113–114. 10.1016/j.jbiotec.2015.09.022 26410453

[B61] Stamatakis A. (2014). RAxML version 8: a tool for phylogenetic analysis and post-analysis of large phylogenies. *Bioinformatics* 30 1312–1313. 10.1093/bioinformatics/btu033 24451623 PMC3998144

[B62] Tamagnini P. Leitão E. Oliveira P. Ferreira D. Pinto F. Harris D. J. (2007). Cyanobacterial hydrogenases: diversity, regulation and applications. *FEMS Microbiol. Rev.* 31 692–720. 10.1111/j.1574-6976.2007.00085.x 17903205

[B63] Tamura K. Stecher G. Peterson D. Filipski A. Kumar S. (2013). MEGA6: molecular evolutionary genetics analysis version 6.0. *Mol. Biol. Evol.* 30 2725–2729. 10.1093/molbev/mst197 24132122 PMC3840312

[B64] Taylor K. B. (1969). The enzymology of nitrogen fixation in cell-free extracts of *Clostridium Pasteurianum*. *J. Biol. Chem.* 244 171–179. 5773280

[B65] Thauer R. K. Kaster A.-K. Goenrich M. Schick M. Hiromoto T. Shima S. (2010). Hydrogenases from methanogenic archaea, nickel, a novel cofactor, and H_2_ storage. *Annu. Rev. Biochem.* 79 507–536. 10.1146/annurev.biochem.030508.152103 20235826

[B66] Valentine R. C. Mortenson L. E. Carnahan J. E. (1963). The hydrogenase system of *Clostridium pasteurianum*. *J. Biol. Chem.* 238 1141–1144.

[B67] Verhagen M. F. O’Rourke T. Adams M. W. (1999). The hyperthermophilic bacterium, *Thermotoga maritima*, contains an unusually complex iron-hydrogenase: amino acid sequence analyses versus biochemical characterization. *Biochim. Biophys. Acta* 1412 212–229. 10.1016/S0005-2728(99)00062-6 10482784

[B68] Vignais P. M. Billoud B. (2007). Occurrence, classification, and biological function of hydrogenases: an overview. *Chem. Rev.* 107 4206–4272. 10.1021/cr050196r 17927159

[B69] Walker C. C. Partridge C. D. P. Yates M. G. (1981). The effect of nutrient limitation on hydrogen production by nitrogenase in continuous cultures of *Azotobacter chroococcum*. *Microbiology* 124 317–327. 10.1099/00221287-124-2-317

[B70] Walker C. C. Yates M. G. (1978). The hydrogen cycle in nitrogen-fixing *Azotobacter chroococcum*. *Biochimie* 60 225–231. 10.1016/s0300-9084(78)80818-9 667178

[B71] Winkler M. Esselborn J. Happe T. (2013). Molecular basis of [FeFe]-hydrogenase function: an insight into the complex interplay between protein and catalytic cofactor. *Biochim. Biophys. Acta* 1827 974–985. 10.1016/j.bbabio.2013.03.004 23507618

[B72] Yutin N. Galperin M. Y. (2013). A genomic update on clostridial phylogeny: gram-negative spore formers and other misplaced clostridia. *Environ. Microbiol.* 15 2631–2641. 10.1111/1462-2920.12173 23834245 PMC4056668

[B73] Zhang X. Sherman D. M. Sherman L. A. (2014). The uptake hydrogenase in the unicellular diazotrophic cyanobacterium *Cyanothece* sp. strain PCC 7822 protects nitrogenase from oxygen toxicity. *J. Bacteriol.* 196 840–849. 10.1128/jb.01248-13 24317398 PMC3911175

