# Supplementary material for: The Physiological Functions and Structural Determinants of Catalytic Bias in the [FeFe]-Hydrogenases CpI and CpII of Clostridium pasteurianum Strain W5
Source: Front Microbiol. 2017 Jul 12;8:1305. doi: 10.3389/fmicb.2017.01305 (PMC5506873; doi:10.3389/fmicb.2017.01305)
Supplement: Supplementary file 1 [file Data_Sheet_1.DOCX]

Supplementary Material

**The physiological functions and structural determinants of catalytic bias in the [FeFe]-hydrogenases of *Clostridium pasteurianum* strain W5**

**Jesse B. Therien, Jacob H. Artz, Saroj Poudel, Trinity L. Hamilton, Zhenfeng Liu, Seth M. Noone, Michael W. W. Adams, Paul W. King, Donald A. Bryant, Eric S. Boyd, John W. Peters^*^**

*** Correspondence:** Corresponding Author: jw.peters@wsu.edu

# Supplementary Figures and Tables

## Supplementary Figures

**
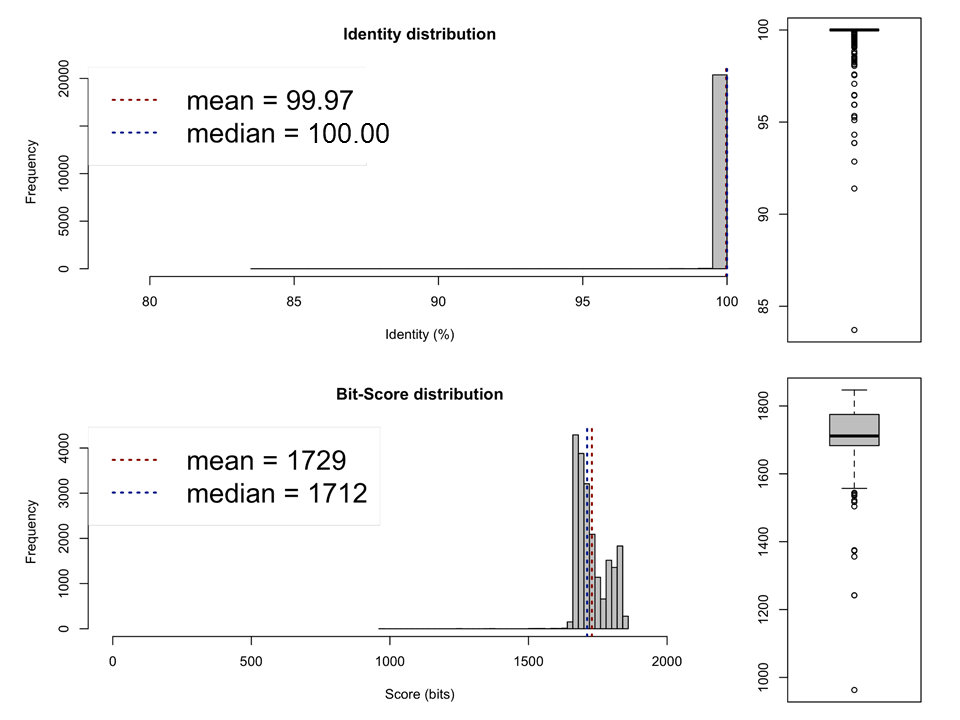
**

**Supplementary Figure 1.** The alignment of the CpW5 genome solved in this work is in near-perfect agreement to the previously sequenced CpW5 genome. [1]

**Supplemental Table 1.**

| Gene | Fwd Primer | Rev Primer |
| --- | --- | --- |
| 16S rRNA | AAGTGAGATGTGAAAGCCCC | AATTCCGCTTTCCTCTCCTG |
| HydAI* | TTGAAGATAGCGAAGCAGACC | CGCCAGTTGCACCAAATATG |
| HydAII* | ACTACCTTCGGAGCAGATTTAAC | TCCACAAATTCTACCCAGGC |
| HydAIII* | CAGAGTTCCCATTGAAAAAGA | ATTGAATAAACAGGGCAAACA |
| HydB* | GTGACACTTGATACAAATAACAATG | GGGATTTACACCTACATTGC |
| NifD | GTCCAGTTGGTCTTATCGGTG | TTGCTATATGGTGACCTGCAG |

*These primers target the large subunit of CpI, CpII, and CpII, and the [NiFe]-hydrogenase, respectively.

**
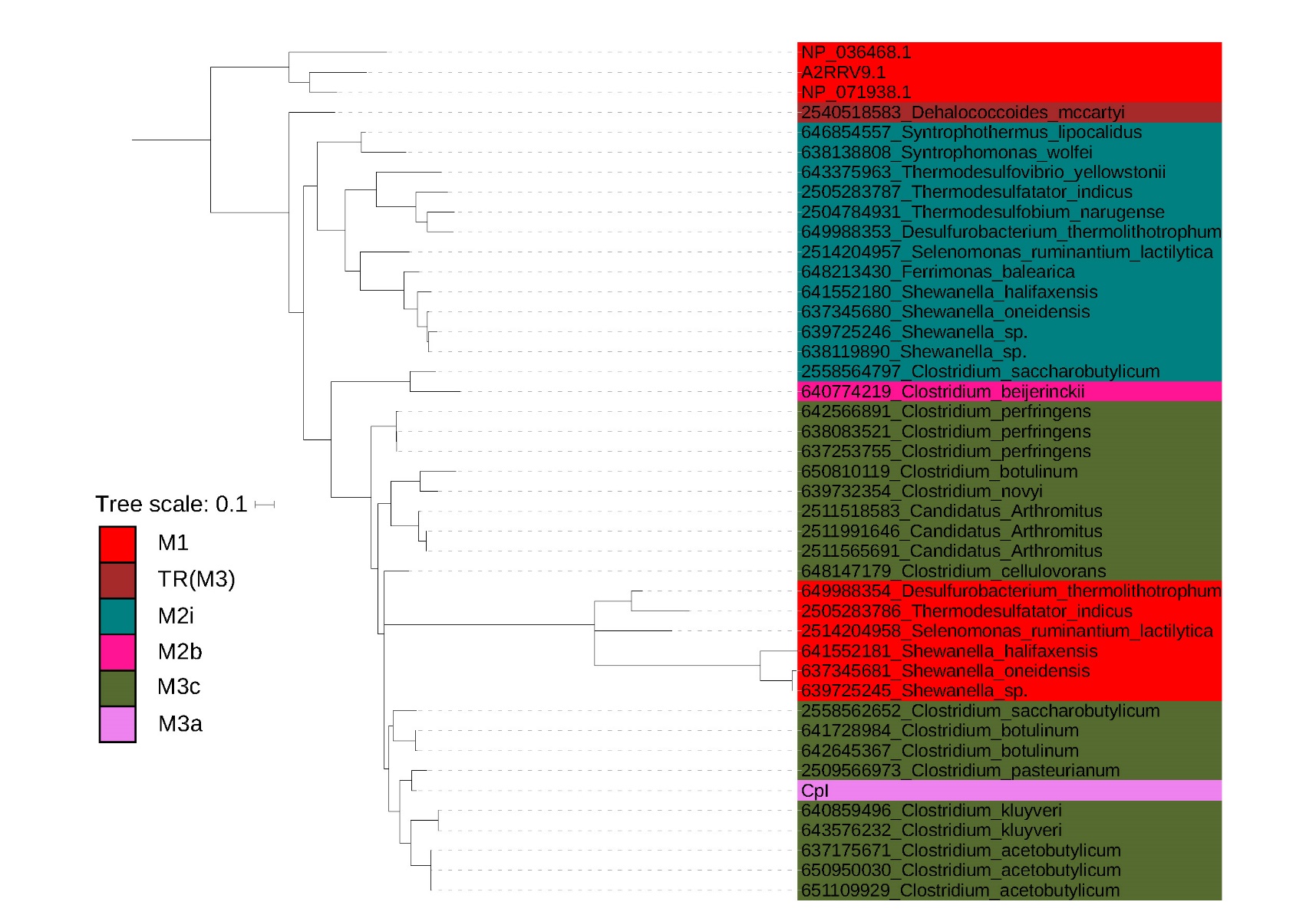
**

**Supplemental Figure 2.**

Hydrogenases that group closely with CpI.

**
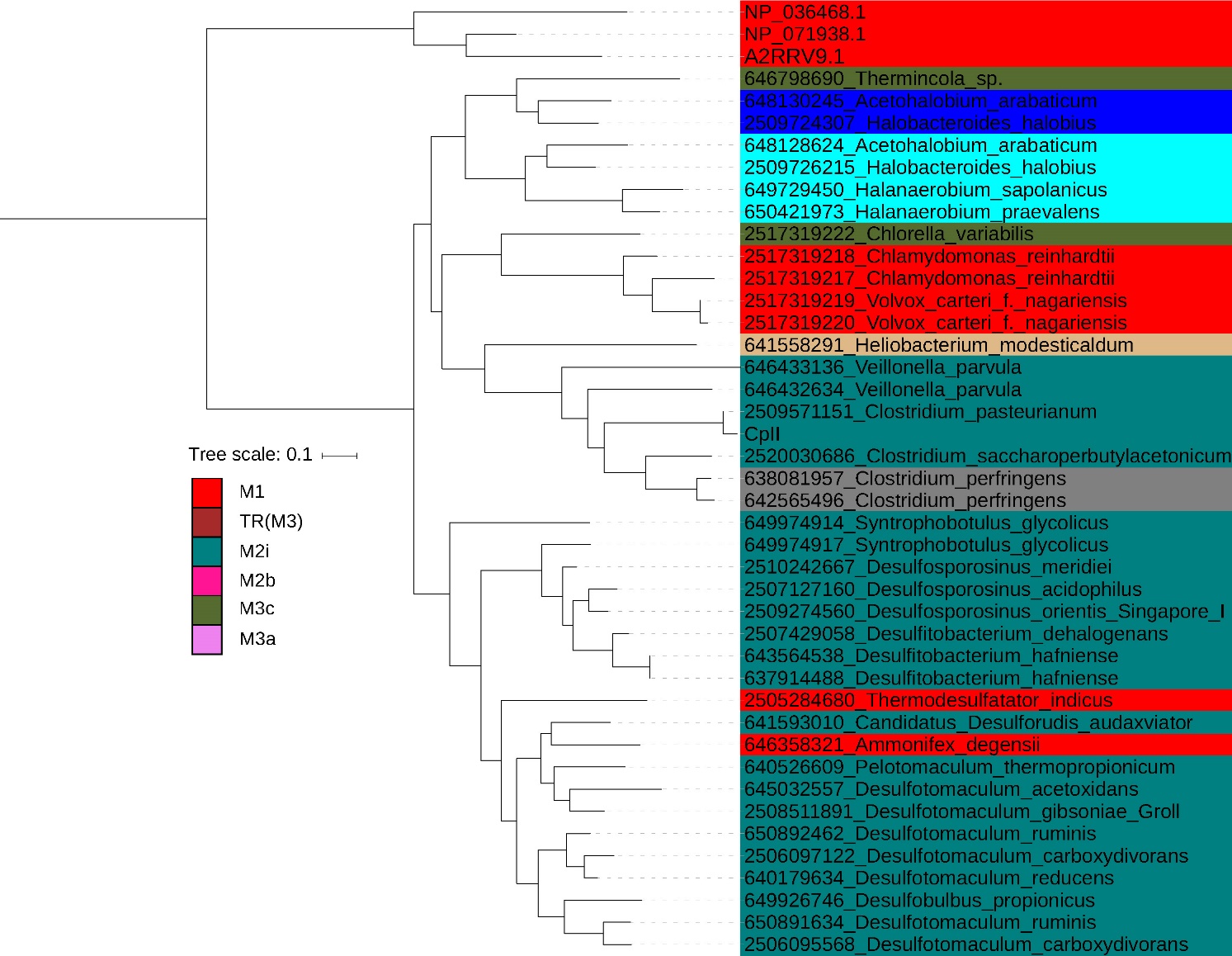
**

**Supplemental Figure 3.**

Hydrogenases that group closely with CpII.

**2.0 References:**

1. Rotta C, Poehlein A, Schwarz K, McClure P, Daniel R, Minton NP: Closed Genome Sequence of *Clostridium pasteurianum* ATCC 6013. *Genome Announcements 2*015, 3: e01596-14.
